# ASARM Mineralization Hypothesis: A Bridge Too Far?

**DOI:** 10.1002/jbmr.69

**Published:** 2010-04

**Authors:** Valentin David, L Darryl Quarles

**Affiliations:** Department of Medicine, Division of Nephrology, University of Tennessee Health Science CenterMemphis, TN, USA

Bone is a dynamic load-bearing organ whose structural integrity is maintained by a remodeling cycle that consists of osteoclast-mediated resorption followed by osteoblast-mediated deposition of an unmineralized collagen matrix (osteoid). After a delay of several days (as measured by the histomorphometric parameter of mineralization lag time), osteoid undergoes mineralization, which is a complex regulated process. Mineralization of osteoid occurs as a consequence of a balance between inorganic factors, such as the local concentrations of phosphate and pyrophosphates, that respectively promote and inhibit hydroxyapatite formation, as well as extracellular matrix proteins that either facilitate or impede the mineralization process. During the mineralization process, a subset of the osteoblasts becomes embedded in the matrix, forming osteocytes that have dendrite-like cytoplasmic extensions creating a canalicular (neural-like) network inside the mineralized matrix, where they act as both sensors and effectors of skeletal homeostasis. This complex regulation of bone turnover and mineralization allows bone to participate in systemic mineral metabolism. In this regard, bone is a mineral reservoir where calcium and phosphate are in equilibrium with the systemic milieu under steady state, and the influx and efflux of calcium and phosphate from bone are under control of both passive physicochemical forces and active cellular processes, such as systemic hormones and mechanical/local factors. A new concept that has emerged is that bone is also an endocrine organ that releases from osteoblasts and osteocytes fibroblast growth factor 23 (FGF23), a novel hormone that targets the kidney to inhibit renal phosphate reabsorption and 1,25-dihydroxyvitamin D [1,25(OH)_2_D] production.(1–4) One physiologic function of FGF23 is to act as a counterregulatory hormone to 1,25(OH)_2_D.(1) The other physiologic function of FGF23 appears to be to serve as the “primary” phosphaturic hormone in a bone-kidney axis that coordinates renal phosphate handling with bone mineralization and possibly bone remodeling activity.(5) There is a major gap, however, in our knowledge of the molecular mechanisms whereby the mineralization process and FGF23 expression are regulated by osteoblasts and osteocytes.

The idea of bone is an endocrine organ that secretes FGF23 to coordinate renal phosphate handling to match bone mineralization and turnover has arisen from the studies of X-linked hypophosphatemia (XLH) and autosomal recessive hypophosphatemic rickets (ARHR).(4,6–9) XLH and ARHR have similar phenotypes, characterized by elevated FGF23 levels, hypophosphatemia, aberrant regulation of 1,25(OH)_2_D production, and rickets/osteomalacia. XLH is caused by mutations of the phosphate-regulating gene *PHEX* with homologies to endopeptidases on the X chromosome, a member of the endothelin-converting enzyme family that leads to an intrinsic defect in bone mineralization and increased *FGF23* gene transcription in osteocytes. ARHR is caused by inactivating mutations of *DMP1*, an extracellular matrix small integrin-binding ligand N-linked glycoprotein (SIBLING protein) that regulates mineralization and is also involved in regulation of transcription of FGF23 by osteocytes.(10) The fact that inactivation of Phex, a cell surface endopeptidase, and DMP1, a SIBLING protein, leads to intrinsic mineralization defects and that these abnormalities of mineralization are associated with elevated FGF23 production by osteocytes might indicate the presence of autocrine/paracrine pathways in bone that coordinate the mineralization process with the production of FGF23, although these pathways have not been clearly defined yet. The alterations in FGF23 by the mineralization process, in turn, regulate renal phosphate handling and 1,25(OH)_2_D production to meet the needs of bone to either increase mineralization (i.e., decrease FGF23) or decrease mineralization (i.e., increase FGF23). The precise mechanism whereby mineralization and FGF23 production are coordinated is not known, but another SIBLING protein called *matrix extracellular phosphoglycoprotein* (MEPE) may play a role in this reciprocal expression. MEPE is not a substrate for Phex, but binds to Phex in a nonproteolytic manner and protects MEPE from proteolytic cleavage by cathepsin B.(11,12)

The article in this issue by Addison and colleagues(13) presents data to support what will be referred to as the *ASARM hypothesis*. This hypothesis is based on the concept that osteoblasts derived from *Hyp* mice produce an unknown secreted factor, called *minhibin*, that inhibits mineralization of extracellular matrix. Minhibin is a theoretical substrate for Phex and would be predicted to accumulate in bone in patients with XLH or the *Hyp* mouse homologue of this disease.(14) MEPE contains a protease-resistant acidic serine-aspartate-rich motif (ASARM peptide) that is a candidate for minhibin.(7) The idea that MEPE is the source of minhibin is derived from the observations that inactivation of *PHEX* in *Hyp* mice is associated with increased proteolytic activity that releases the ASARM peptide that accumulates in the extracellular matrix to inhibit the mineralization process. In addition, this ASARM peptide appears to be degraded by Phex, which further contributes to its accumulation(15) in the absence of Phex. Other studies demonstrate that anti-ASARM antibodies or soluble Phex-derived peptides sequestrate ASARM and correct the defective mineralization of *Hyp-*derived osteoblasts and bone marrow stromal cells (BMSCs) in vitro.(16) The relevance of MEPE to bone is also supported by mapping of a bone mineral density loci in humans to 4q21.1, a region where the *MEPE* gene is located in humans(17) and the small age-dependent increase in bone density in *MEPE* null mice (18). Finally, *MEPE* overexpression in mice, under the control of the *Col1a1* promoter, leads to increased MEPE and ASARM levels in bone and defective mineralization, and this would appear to support the ASARM hypothesis.

The ASARM motif is also present in other SIBLING proteins, including DMP1 and osteopontin. Recent studies,(16) including the current JBMR report,(13) suggest that ASARM peptides derived from other SIBLINGs proteins also may regulate the mineralization process. Since the OPN ASARM motif shares 60% homology with MEPE ASARM, the investigators examined if the OPN ASARM peptide inhibits mineralization in vitro. Similar to what was previously reported for the MEPE ASARM, Addison and colleagues found that the phosphorylated OPN ASARM was cleaved by Phex and that the addition of this phosphorylated peptide to osteoblast cultures inhibited mineralization in vitro.(13) They also presented evidence that the degree of phosphorylation of ASARM also affected its hydroxyapatite binding and mineralization inhibitory activity. Indeed, tri- and pentaphosphorylated OPN ASARM inhibited mineralization in vitro, the peptide containing 5 phosphates being more potent, whereas unphosphorylated ASARM peptides had no effect on crystal apposition and growth. Only triphosphorylated OPN ASARM-mediated inhibition of mineralization could be prevented by the addition of a soluble recombinant Phex to the osteoblast cultures, whereas Phex failed to rescue pentaphosphorylated ASARM mineralization inhibition.

Is this ASARM hypothesis true, or is it an example of an amalgamation of in vitro findings that have lead to a false model for in vivo mineralization? The answer to this question is not yet known, but the known differences in the in vivo functions of ASARM containing SIBLING proteins and the proposed common in vitro effects of the ASARM peptide derived from these SIBLING proteins should lead to a further review of this hypothesis. Indeed, there are several discordant findings that need to be addressed before accepting the ASARM peptide model.

First, the phenotypes of *MEPE*, *DMP1*, and *OPN* null mice do not precisely fit with the ASARM hypothesis. For example, the bone phenotype of *MEPE* null mice, which would lack ASARM derived from MEPE, is almost imperceptible, which is inconsistent with a major role of ASARM in regulating bone mineralization. In addition, in *Hyp* mice, where increased ASARM peptide derived from MEPE is believed to be the major mineralization inhibitor, the ablation of MEPE by crossing *MEPE* null mice onto the *Hyp* background fails to rescue the rickets and osteomalacia in vivo.(18) Additionally, in vivo conditional osteocalcin-promoted (OC-promoted) *PHEX* inactivation, while leading to rickets and osteomalacia, a phenotype similar to *Hyp* mice, presents a massive reduction in MEPE(19) and MEPE ASARM peptides.(20) Other alterations in *MEPE* transgenic mice, such as hyperphosphatemia, and increased *PHEX* expression, coupled with the fact that phosphate restriction corrected most of the osseous abnormalities in *MEPE* transgenic mice, suggest that MEPE and/or ASARM may not function as inhibitors of mineralization under all conditions.(21) The biologic relevance of the in vitro finding that the related ASARM peptide in OPN has similar functions to the MEPE-derived peptide also remains uncertain. As pointed out, mice deficient in OPN do not have defective mineralization and have skeletal patterning indistinguishable from that of control mice.(22–24) The idea that redundant sources of ASARM from other SIBLINGs might collectively contribute to inhibition of mineralization also lacks support because no data have yet shown production of ASARM peptides from other SIBLINGs. *DMP1* has an ASARM motif, but ablation of *DMP1*, and loss of its ASARM peptide, leads to impaired mineralization. No studies have shown that the loss of ASARM peptide generation leads to enhanced mineralization, as the ASARM peptide hypothesis would predict. Use of mouse genetics to create compound deletions of SIBLING proteins to address the issue of redundancy, however, is difficult because the genes encoding SIBLINGS are closely grouped on the same chromosome. Regardless, the overall function of individual SIBLING proteins defined by mouse genetic approaches does not support a conserved function of the embedded ASARM motif to act as biologically relevant inhibitor of mineralization. However, the ASARM motif could have a conserved function that is yet to be defined.

Second, native proteins (with ASARM motifs) and synthetic ASARM peptides appear to alter mineralization differently,(25) and the structure of endogenous fragments is still unknown. Moreover, the requirement for phosphorylation of ASARM on serine residues for inhibition of mineralization in vitro has not been demonstrated in vivo. Casein kinase II is an enzyme that might phosphorylate the ASARM peptides; however, in *Hyp* mice the activity of casein kinase II is diminished.(26) Thus it is not clear that phosphorylation of ASARMs occurs in vivo, at least in XLH. In addition, the specificity of the response is uncertain because there were no controls reported using other phosphorylated peptides that might also inhibit mineralization under similar in vitro conditions. The ability of other acid peptides to inhibit mineralization would weaken the significance of the existing in vitro data and make confirmation of the function of ASARM in vivo even more relevant.

Finally, there are also spatial constraints to the notion that the ASARM peptide is both a substrate for Phex and inhibitor of mineralization by binding to hydroxyapatite crystals in extracellular matrix. In this regard, Phex is a membrane bound endopeptidase located in osteocytes and osteoblasts, whereas the actions of ASARM require binding to hydroxyapatite crystals in the extracellular matrix that is distant from the osteoblast surface. It is not clear how, under normal steady-state conditions, where intact ASARM and cleaved peptides are in equilibrium, hydroxyapatite-bound ASARM peptide is trafficked from collagen to the cell surface to be degraded by Phex. However, other mechanisms could be theorized, such as the presence of Phex-containing “nanospherulites” that could permit Phex to function at sites of active mineralization. If the ASARM hypothesis is correct, new investigations should explore how Phex might localize to the mineralization microenvironment that is spatially separated from the osteoblast membrane surface. Another important notion is whether or not Phex cleaves the ASARM motif from native proteins because there seems to be no prerequisite for phosphorylation.(13) Moreover, increments in serum phosphate can almost completely cure rickets and osteomalacia in patients with XLH without prior removal of the ASARM peptides, although no data are available to confirm ASARM sustained production.

Thus, while phosphorylated OPN-derived ASARM peptides may inhibit mineralization in culture or inhibit the growth of calcium oxalate monohydrate crystals in vitro, this does not necessarily mean that the physiologic function of OPN or the ASARM peptides derived form OPN is to regulate the mineralization process. Nevertheless, the in vitro actions of phosphorylated ASARM peptides to inhibit mineralization are interesting, and a physiologic function of phosphorylated ASARM peptides is not a prerequisite for these peptides to be a potential therapeutic biologic agent. However, further work is needed to establish the role of ASARM peptide production and metabolism in regulating bone mineralization.
